# Drosophila as a Model for Developmental Biology: Stem Cell-Fate Decisions in the Developing Nervous System

**DOI:** 10.3390/jdb6040025

**Published:** 2018-10-19

**Authors:** Katherine Harding, Kristin White

**Affiliations:** Massachusetts General Hospital Cutaneous Biology Research Center, Harvard Medical School, Boston, MA 02129, USA

**Keywords:** stem cell biology, neuroblast, cell cycle, apoptosis, *Drosophila*, development

## Abstract

Stem cells face a diversity of choices throughout their lives. At specific times, they may decide to initiate cell division, terminal differentiation, or apoptosis, or they may enter a quiescent non-proliferative state. Neural stem cells in the *Drosophila* central nervous system do all of these, at stereotypical times and anatomical positions during development. Distinct populations of neural stem cells offer a unique system to investigate the regulation of a particular stem cell behavior, while comparisons between populations can lead us to a broader understanding of stem cell identity. *Drosophila* is a well-described and genetically tractable model for studying fundamental stem cell behavior and the mechanisms that underlie cell-fate decisions. This review will focus on recent advances in our understanding of the factors that contribute to distinct stem cell-fate decisions within the context of the *Drosophila* nervous system.

## 1. Introduction

Control of stem cell divisions is critical for normal organismal development. The overall proliferative potential of a given tissue is defined by several parameters, including the total number of stem cells, temporal control of proliferation and capacity to divide, and longevity within the tissue. The generation of progeny cells with specific differentiation programs must be coordinated in both time and space for proper tissue organization. Accurate transmission of identity factors from stem cell to progeny is therefore also critically important for organogenesis. Unregulated proliferation of cells with stem-like behavior is commonly associated with tumorigenesis and can lead to metastases.

**Figure 1:**
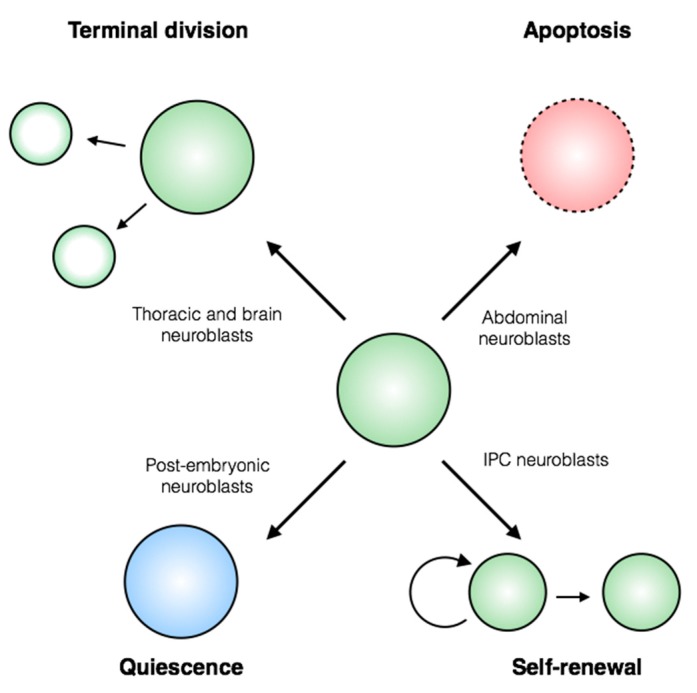
**Stem cell-fate decisions in the *Drosophila* central nervous system.***Drosophila* neural stem cells, called neuroblasts, exhibit diverse stem cell behaviors. Thoracic and brain neuroblasts are eliminated by terminal division (top left), while abdominal neuroblasts undergo apoptosis (top right). Temporal controls regulate the period of neuroblast quiescence between embryonic and larval stages of development (bottom left). Certain larval neuroblasts within the inner proliferating center (IPC) of the optic lobe switch from renewing asymmetric to amplifying symmetric divisions (bottom right). Execution of these cell-fate decisions is intimately tied to neuroblast identity in time and space.

**Figure 2:**
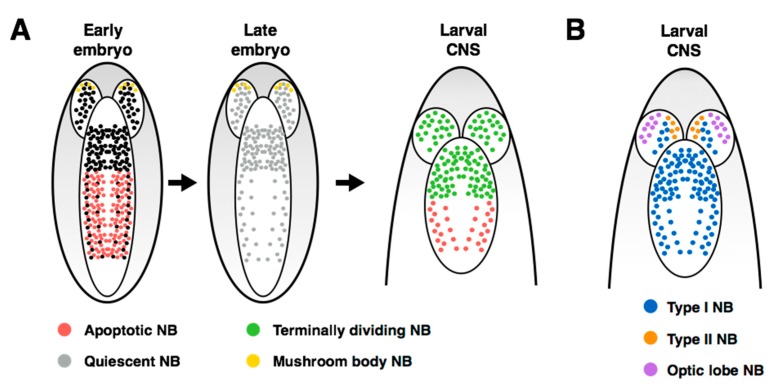
**Neuroblast populations within the *Drosophila* central nervous system.** (**A**) Cell-fate decisions are mapped onto anatomical populations of neuroblasts during embryonic and larval development. The majority of abdominal neuroblasts undergo apoptosis during embryogenesis, leaving 3 neuroblasts per hemisegment in the larval CNS. Neuroblasts stop proliferating between embryonic and larval development, except for 4 mushroom body neuroblasts. Following reactivation, neuroblasts in the thorax and brain undergo terminal division, while the remaining abdominal neuroblasts die through apoptosis. (**B**) Proliferation patterns of neuroblasts throughout the larval CNS. Most neuroblasts use the Type I division pattern, except a small population of Type II neuroblasts in the brain. The neuroblasts in the inner proliferating center of the optic lobe have been observed to divide symmetrically.

**Figure 3:**
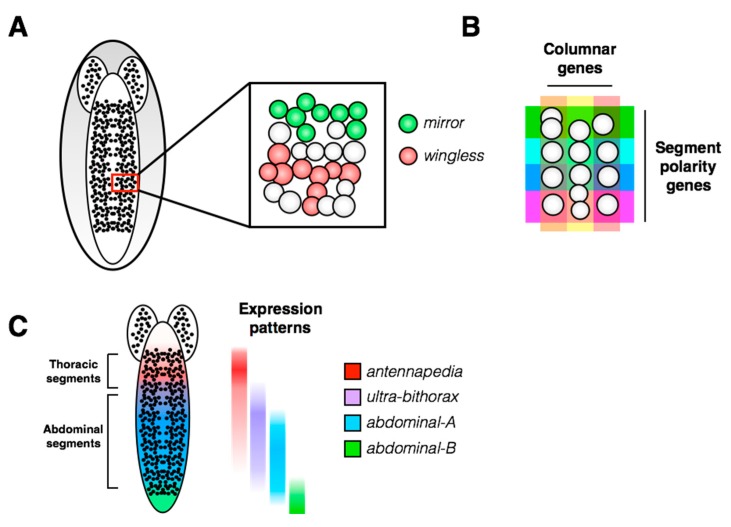
**Spatial patterning of the *Drosophila* ventral nerve cord.** (**A**) A single hemisegment containing approximately 30 neuroblasts is shown in the inset. Gene expression is patterned within the hemisegment: two examples of spatially restricted molecular markers are shown (*mirror* and *wingless*; from the Hyper-Neuroblast Map, Doe laboratory). (**B**) Upon delamination from the neuroectoderm, neuroblast identity is specified within a hemisegment by a Cartesian grid generated by expression patterns of the columnar and segment polarity genes (reviewed in [16]). (**C**) Along the anterior-posterior axis of the embryo, hemisegment identity is also subject to spatial regulation by the Hox gene family. All of these spatial patterns are superimposed *in vivo*, providing each neuroblast with a unique spatial identity.

**Figure 4:**
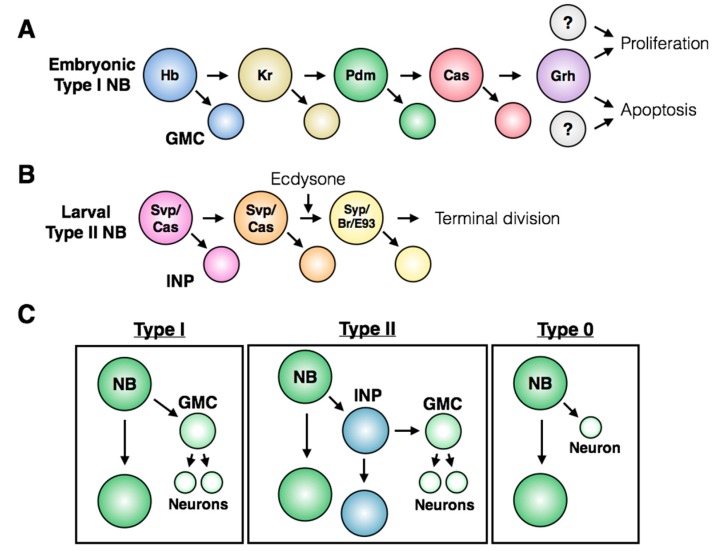
**The temporal series and division patterns of *Drosophila* neuroblasts.** (**A**) The temporal transcription factor (TTF) sequence expressed in embryonic Type I neuroblasts. The last member, *grainyhead*, has been shown to regulate both proliferation and apoptosis of neuroblasts, suggesting that context-dependent co-factors may dictate the cell-fate outcome for a given neuroblast. (**B**) The TTF sequence expressed in larval Type II neuroblasts. Progression to the late factors is dependent on the steroid hormone ecdysone, and leads to a terminal division [26,32]. (**C**) Three types of asymmetric neuroblast proliferation patterns observed in the nervous system. Type 0 divisions have only been observed following a period of Type I divisions in embryonic neuroblasts.

**Figure 5:**
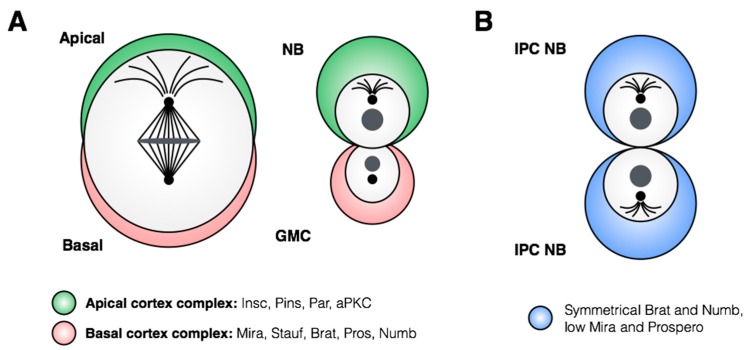
**Localization of proteins during asymmetric and symmetric neuroblast divisions.** (**A**) A typical asymmetric neuroblast division involves segregation of apical and basal cortex complex members to opposite sides of the dividing neuroblast. Proteins within the apical cortex complex are retained in the stem cell, while factors associated with the basal cortex complex are transmitted to the daughter GMC. (**B**) Symmetric divisions of the IPC neuroblasts display low Prospero and Miranda protein levels, but Brat and Numb proteins are divided equally between the two new stem cells [51].

In this review, we will discuss the current understanding of the mechanisms that control stem cell-fate decisions in the *Drosophila melanogaster* central nervous system (CNS). Neural stem cells, called neuroblasts, are present throughout the *Drosophila* CNS, and generate both neurons and glia as progeny. The biology of these stem cells has been sufficiently characterized to begin to identify molecular differences between neuroblast populations, as well as heterogeneous behavior within a given population. Among these differences are proliferation patterns, progeny identities and terminal fate decisions (Figure 1). Stem cell populations are traditionally defined by their anatomical position, and a vast body of work has described spatial control of stem cell proliferation and terminal fate (Figure 2; [1,2,3,4,5]). In addition, neurogenesis occurs in several waves throughout the development of the fly, allowing temporal control of stem cell decisions to be examined. We summarize the strengths of the *Drosophila* CNS as an easily accessible, genetically tractable model to study fundamental processes of stem cell biology. 

## 2. Overview of the Fly Nervous System and Its Stem Cell Populations

The *Drosophila* nervous system is shaped in stages: the distinct phases of neurogenesis and tissue sculpting correspond to the distinct innervation requirements of the animal as it develops from a crawling larva to an adult fly. *Drosophila* neurogenesis begins in the embryo with neuroblast delamination from the neuroectoderm. Delamination and acquisition of neuroblast identity depends on Notch signaling interactions between adjacent neuroectodermal cells, leading to stem cell-specific expression of pro-neural genes (reviewed in [6]). Neuroblast specification from the neuroectoderm is also dependent on the Sox genes *SoxN* and *Dichaete*, and members of the Snail family (*snail*, *worniu*, and *escargot*). Dichaete and SoxN act redundantly in the neuroectoderm to promote neuroblast identity through pro-neural gene expression [7,8,9,10,11]. Loss of Snail family function does not prevent neuroblast delamination, but expression of the pro-neural genes is inhibited and CNS development is severely impaired [12,13,14].

Neuroblast delamination occurs along the ventral side of the embryo and from neural placodes at the head of the embryo, leading to the development of the ventral nerve cord (VNC) and brain, respectively. The embryonic VNC consists of repeated structural units called segments, which are divided by the midline of the embryo into hemisegments, each containing 30 unique neuroblasts (Figure 3A). Segmental identity differs along the anterior-posterior axis of the embryo: there are 3 anterior gnathal segments, 3 thoracic segments and 10 abdominal segments, followed by the non-segmented telson at the posterior end of the VNC. In addition to the anterior-posterior positional information at the whole animal level, there is a positional map within each hemisegment of the VNC. Patterned expression of positional genes creates a Cartesian grid in which each of the 30 neuroblasts can be assigned a unique identity based on its molecular markers (Figure 3B, [15,16]). Transplantation and *in vitro* explant studies have shown that neuroblast molecular identity is defined early in embryogenesis and is retained cell-autonomously [17,18,19,20,21]. The spatial information within each hemisegment is superimposed upon the anterior-posterior axis, where positional information is provided by the Hox gene family (Figure 3C). The segmental pattern of molecular markers seems invariant along the anterior-posterior axis [22]. Birkholz et al. (2013) also determined that, while the terminal abdominal segments contain many fewer neuroblasts than more anterior abdominal segments, the spatial patterns of gene expression are retained in the remaining neuroblasts.

Along with positional identity, neuroblasts can also be temporally defined by the expression pattern of sequential markers, known as the temporal series or temporal transcription factors (TTFs; [23,24,25,26,27]). Figure 4A illustrates the TTF series for embryonic neuroblasts found in the thorax and abdomen, while Figure 4B shows the series for a subset of larval neuroblasts within the central brain. The TTFs confer temporal identity to neurons born within each TTF window, and their cross-regulation ensures unidirectional progression through the series. Many fate decisions lie downstream of the TTFs, including terminal division and induction of apoptosis [4]. While the exact TTF series differs between specific neuroblast populations, the use of cross-regulatory transcription factors to confer temporal identity to neuroblasts and their progeny is found throughout the nervous system. 

Neuroblast identity, in both time and space, fundamentally underlies the behavior and fate of the stem cell. For example, neuroblast proliferation patterns differ between populations of stem cells, with three major division types occurring throughout the CNS (reviewed in [28]). Within the thorax and abdomen of the embryonic VNC and the central brain of the larval CNS, most stem cell divisions are asymmetric and produce a new stem cell and a smaller ganglion mother cell (GMC). The GMC will then divide to produce two daughter cells, which terminally differentiate into neurons or glia. This pattern of division is referred to as a Type I division (Figure 4C, left). A subset of neuroblasts in the larval central brain undergo Type II divisions, in which the neuroblast asymmetric division produces a stem cell and an intermediate neural progenitor (INP) that matures prior to dividing asymmetrically to produce another progenitor and a GMC (Figure 4C, middle; [29,30,31]). Type II neuroblasts have only been described within the brain lobes: their presence in this region may reflect the high proliferative capacity required to generate a complex CNS. If the central brain contained only Type I neuroblasts, it is tempting to speculate that the limited proliferative potential of this type of stem cell division would prevent the production of diverse types of neurons and supporting cells required for higher level processing. 

An alternative mode of division has also been described within the embryonic VNC, called a Type 0 division: the asymmetric division of the neuroblast produces a stem cell and a terminally differentiated neuron, without a GMC intermediate (Figure 4C, right; [33,34,35]). In certain cases, a single neuroblast will use multiple proliferation patterns throughout its lifetime. For example, embryonic neuroblasts in the thorax and abdomen undergo a Type I > 0 switch towards the end of embryogenesis [33]. Neuroblasts have only been observed to use the Type 0 pattern following a period of Type I divisions, so regulation of this switch by temporal series members seems to be the critical inducer of Type 0 neuroblast divisions.

The molecular machinery regulating asymmetrical neuroblast divisions has been well characterized in *Drosophila*, and has been covered in depth elsewhere [36,37]. Briefly, the asymmetrical division depends on accurate spindle orientation along the apical-basal axis of the neuroblast, mediated by the apical Par/aPKC complex (Figure 5A). In addition to spindle orientation, this complex regulates subcellular localization of cell-fate determinants, which accumulate at the basal cortex of the dividing neuroblast and are thus transmitted only to the GMC. The scaffold protein Miranda plays a key role in asymmetrical localization of regulators of cell fate, including the mRNA-binding protein Staufen, which is responsible for sequestering transcripts for the transcriptional regulator and pro-differentiation factor Prospero [38,39,40,41,42,43,44]. Disruption of correct spindle orientation through loss of function of the Par/aPCK complex causes misalignment of the spindle and prevents proper segregation of cell-fate determinants, leading to accumulation of stem cell-like progenitors throughout the nervous system [45,46,47,48,49,50].

## 3. Control of Stem Cell Proliferation: Self-Renewal and Amplification 

The overall number of stem cells within a tissue affects its proliferative potential by limiting the maximum number of progeny that can be produced simultaneously. The generation of tissue-specific stem cells from multipotent cells must therefore be carefully coordinated to ensure proper downstream progeny numbers and organization. As described above, the production of stem cells from the embryonic neuroectoderm is limited by Notch-mediated lateral inhibition between a newly born neuroblast and its neighbors [52,53,54]. The embryonic phase of neuroblast delamination generates ~30 neuroblasts per thoracic or abdominal hemisegment in the VNC, and ~105 neuroblasts in each central brain lobe. Historically, the ~800 neuroblasts of the optic lobe were thought to be generated from the neuroepithelial layer during larval development [55]. However, a recent study observed the generation of embryonic optic neuroblasts (EONs) from the neuroepithelium overlying the developing embryonic brain [56]. This study indicates that optic lobe development begins much earlier than previously thought and provides an additional system for studying the coordination of embryonic and larval neurogenesis.

Until recently, the number of stem cells within the nervous system was thought to be defined by the number of delaminated neuroblasts, as all neuroblasts were only observed to divide asymmetrically. This model was challenged by a recent study that demonstrated that neuroblasts of the inner proliferating center (IPC) of the larval optic lobe undergo a temporal switch to symmetric amplifying divisions [51]. Unlike dividing Type I/II neuroblasts, the symmetric divisions of the larval IPC neuroblasts do not show asymmetric segregation of Prospero, Mira or Brat proteins (Figure 5B; [51]), indicating a fundamentally distinct mechanism of neuroblast division in these cells. This division pattern is induced by the pro-neural gene *atonal*, but the authors also observe that *atonal* maintains neuronal differentiation. It is therefore unclear which *atonal* targets contribute to the induction of symmetric neuroblast divisions in the IPC. As *atonal* is a pro-neural gene required for neuroblast identity, and symmetric divisions have only been observed in this limited population, context-dependent pathways are likely to play a role in promoting symmetric divisions. 

With respect to the generation of Type I or Type II neuroblasts in the embryo, the upstream signals that direct stem cells to a particular division pattern remain unclear. Close characterization of TTF expression in each type of neuroblast demonstrates that they progress through distinct temporal identities (reviewed in [25]). Much of the work investigating TTF expression in neuroblasts has focused on the generation of neural diversity through transmission of temporal identity to progeny. It is unclear whether the TTF series themselves direct neuroblast proliferation patterns, or if expression of these transcription factors lies downstream of the Type I vs II decision. However, the switch during embryogenesis from Type I > 0 does lie downstream of TTFs: loss of function of *castor* or *grainyhead*, both late TTFs, results in extra neuroblast and daughter proliferation in the embryo [33]. Two recent studies showed that central brain Type II neuroblasts arise during embryogenesis, and identified the epidermal growth factor (EGF) pathway as an upstream determinant of the Type II identity [57,58]. EGF signaling pathway components, including the EGF receptor ligand Spitz, the Rhomboid protease required for ligand release and the downstream transcription factor Pointed P1 (PntP1) are required for embryonic specification of Type II neuroblasts [57,58]. Embryonic Type I neuroblasts are PntP1-negative [59,60], suggesting that EGF signaling is a positive driver of the Type II identity. Another outstanding question relates to whether neuroblasts can be induced to switch proliferation patterns following delamination, or if the Type I/II decision is an immutable quality determined by hemisegment identity and anterior-posterior position. It was recently shown that loss of function of *inscuteable* (Insc), a regulator of cell-fate determinant localization, results in INP-like progenitor cells within larval Type I lineages [61]. These findings begin to shed light on the question of how Type I and Type II neuroblasts are distinguished prior to the onset of their distinct TTF series and implicates the upstream regulators EGF and Insc in the Type I/II choice. 

The previous examples described differences in the fundamental proliferation patterns of specific neuroblast populations. However, the division capacity and identity of progeny of individual neuroblasts within a population can differ widely across the anterior-posterior axis of the nervous system, even between stem cells that share the same identity in a hemisegment [18,20,62,63,64,65,66]. For example, during embryogenesis the thoracic neuroblast NB1-1 produces multiple neuronal types but no glia, while the abdominal NB1-1 produces fewer neurons and 3 glia [18]. Spatial information conferred by Hox gene expression is sufficient to induce changes in division capacity: ectopic expression of the Hox genes *ultrabithorax* or *abdominal-A* in the thoracic NB1-1 resulted in the production of glial progeny [18]. Berger et al. (2005) [67] demonstrated that transformation of thoracic NB6-4 by AbdA (as determined by progeny output) requires CycE, and that CycE expression was sufficient to induce the positional transformation. As other cell cycle manipulations did not produce this effect, the role of CycE in segmental identity was proposed to be independent of its function in the cell cycle. Indeed, the same group determined that these two functions of CycE are mediated through distinct protein domains, and that CycE both promotes the cortical localization of Prospero protein, and interferes with Pros-mediated gene regulation [68]. More recently, the upstream factors *glial cell missing* (gcm) and *apontic* (Apt) were found to be differentially expressed between thoracic and abdominal NB6-4, resulting in restriction of CycE expression to the thoracic NB6-4 [69]. These findings provide the most direct evidence for how Hox-mediated differences in segmental identity influence neuroblast proliferation behavior. 

## 4. Regulation of Stem Cell Quiescence 

As well as a model for stem cell proliferation, neuroblasts offer a tractable system to study the regulation and maintenance of endogenous stem cell quiescence. Embryonic neuroblast divisions were meticulously described by Hartenstein et al. (1987) [70], and the lull in neuroblast proliferation at the end of embryogenesis was first described thirty years ago (Figure 3A, middle; [1]). Since that time, a large body of work has characterized the timing and regulation of the reactivation of proliferation in the larval nervous system. With the exception of four mushroom body neuroblasts that do not enter quiescence ([71], Figure 3A), the neuroblasts in the central brain are the first to begin re-proliferating, within 24 h after larval hatching (ALH; [1,71,72]). This is followed by reactivation of the thoracic neuroblasts at ~30h ALH, and then the abdominal neuroblasts after ~50h ALH. The mechanisms regulating the anterior-posterior gradient of neuroblast proliferation have been recently investigated by the Thor lab. They observe that the lineage size of a neuroblast is regulated by Polycomb group proteins (PcG), whose members inhibit Hox gene expression in the brain and thereby promote continued neuroblast proliferation in this region of the nervous system [65,66]. While these studies establish a role for PcG and Hox genes in the regulation of neuroblast proliferation, the mechanism by which they control neuroblast reactivation following quiescence remains unclear.

**Figure 6:**
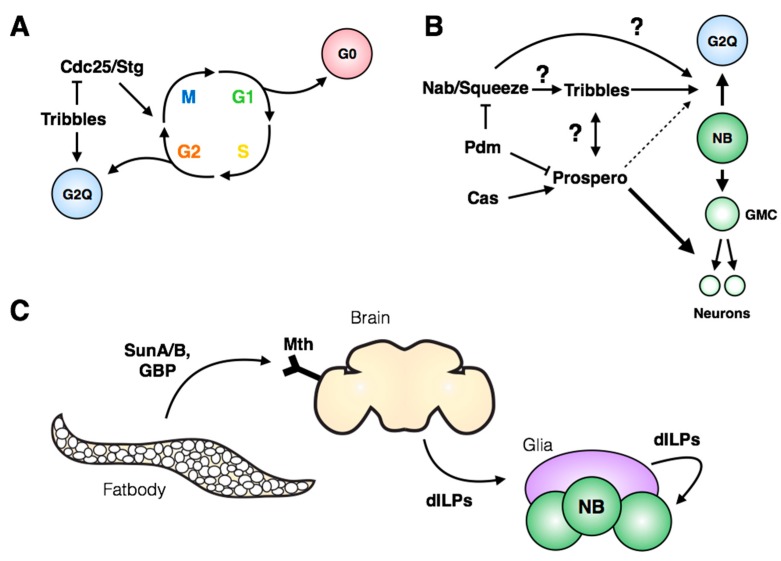
**Regulation of neuroblast quiescence and reactivation.** (**A**) The majority of post-embryonic neuroblasts exit the cell cycle at G2 phase (G2Q), while the remaining ~25% display a canonical G0 arrest [73]. Tribbles promotes and maintains quiescence through degradation of Cdc25/Stg and inhibition of Akt signaling (not shown). (**B**) Prospero regulates neural differentiation and is required for neuroblast quiescence, but the mechanism underlying its ability to induce two different cell fates is unclear. The connections between G2Q and the Prospero regulatory network are also unknown. (**C**) Secreted factors from the larval fatbody are sensed by the brain, leading to secretion of insulin-like peptides (dILPs). dILPs are also expressed by stellate surface glia, and these act on the neuroblast to promote growth and proliferation.

Despite three decades having passed since the first description of neuroblast quiescence, it was only recently recognized that three quarters of post-embryonic neuroblasts exit the cell cycle in G2 (Figure 6A; [73]), while the remaining 25% display a more standard G0 quiescence. The authors identified the pseudokinase *tribbles* as a major regulator of G2 quiescence entry and stem cell reactivation (Figure 6A). Tribbles has been shown previously to mediate degradation of Cdc25/String [74,75,76], leading to maintenance of inhibitory phosphorylation on Cdk1 and preventing cell cycle progression. In addition to its role in inducing quiescence, Tribbles maintains neuroblast quiescence into larval life by inhibiting Akt activation [73]. Interestingly, the authors demonstrate that G2-arrested stem cells are reactivated more quickly in the larval nervous system compared to G0-arrested stem cells. This finding opens many questions regarding the significance and regulation of G2 quiescence: how broadly is G2 quiescence used in other stem cells in flies or mammals? What molecular signatures distinguish G2-quiescent cells from the G2 phase of actively cycling cells? Further work will be needed to link these newest observations with the previous body of work describing spatial and temporal regulation of neuroblast entry into quiescence.

The transcriptional regulator Prospero has been well characterized as an inhibitor of proliferation in the nervous system. Prospero is suggested to act as a “binary switch” between neural stem cells and differentiated neural progeny: targets of Prospero include genes involved in cell cycle regulation, neuroblast identity, the temporal series and neuronal differentiation [77]. In addition to its role in differentiation, Prospero has also been implicated in neuroblast quiescence, as embryonic neuroblasts express a pulse of transient nuclear Prospero that is required for their quiescence (Figure 6B; [78]). Inhibition of embryonic temporal series progression with *pdm* and *castor* mutants did not prevent quiescence, indicating that, while the timing of quiescence is shifted, the mechanism leading to accumulation of nuclear Prospero is independent of the temporal series [78]. In light of the recent work from the Brand lab [73], it is of strong interest to determine how nuclear Prospero could lead to reversible G2 quiescence in neuroblasts at the end of embryogenesis, while promoting differentiation in neuronal progeny (Figure 6B). Differences in target gene sensitivity to levels in Prospero protein may be involved: a threshold model was introduced by Lai & Doe (2014) [78] to explain differential repression of target genes. The threshold model could encompass both contexts in which target gene expression is solely dictated by Prospero levels, and situations in which Prospero cooperates with co-factors whose expression patterns dictate the identity and amplitude of target gene expression. An additional layer to this model is the possible regulation of co-factors by Prospero itself. 

Context-specific regulation of quiescence entry can be examined by comparing neuroblasts across anatomical regions of the VNC. The timing of quiescence entry differs for a given embryonic neuroblast between the thorax and abdomen [79], indicating that both temporal and spatial signals influence the mechanisms regulating entry into quiescence. The Hox gene *antennapedia* (Antp) is required for quiescence of the thoracic NB3-3, while *abdA* expression is sufficient to induce extended proliferation of thoracic NB3-3, copying the endogenous division pattern of the abdominal NB3-3 [79]. Perhaps intuitively, the quiescent state itself prevents progression through the temporal series: continued expression of the late temporal series members Castor and Grainyhead ensures a retained memory of the temporal identity of the neuroblast upon cell cycle re-entry [79]. However, the molecular details of this inhibition are unclear. The regulation of embryonic neuroblast quiescence at the spatial and temporal level should ultimately coincide with the induction of a transient Prospero pulse and the expression of Tribbles in post-embryonic neuroblasts (Figure 6B). Tsuji et al. (2008) placed the transcription factors Nab and Squeeze downstream of the temporal series and showed their necessity for quiescence entry. It remains to be tested whether Nab and Squeeze represent the intermediaries to Prospero and Tribbles induction (Figure 6B), and whether their own expression is under control of spatial factors as well as the temporal regulation described in Tsuji et al. (2008). The reversible quiescence of post-embryonic neuroblasts represents a mechanism of cell cycle exit fundamentally distinct from Prospero-mediated terminal differentiation of neuronal progeny, and much remains to be uncovered with respect to its regulation and molecular signatures.

## 5. Induction of Neuroblast Cell Cycle Re-Entry

Following the period of post-embryonic quiescence, neuroblasts are reactivated in the larval nervous system for a second wave of proliferation. Cell cycle re-entry (from both G2 and G0 quiescence) is induced by nutrient-sensing pathways that relay environmental signals to the nervous system, promoting growth and continued development (Figure 6C). In the absence of amino acid intake, larval neuroblasts do not reinitiate proliferation [72,80,81]. The mushroom body neuroblasts, which do not enter quiescence, proliferate independently from amino acid intake due to continued expression of the transcription factor Eyeless [82,83]. Investigations into the signaling pathways that transmit nutritional signals to neuroblasts have provided a framework for studying the role of the stem cell niche on stem cell proliferation.

Early studies identified a positive role for glia in the regulation of post-embryonic neuroblast quiescence: glial cell secretion of the glycoprotein *anachronism* (Ana) is required for maintaining quiescence, while the fly Perlecan homologue, *terribly reduced optic lobes* (Trol), promotes proliferation downstream of *ana* [84,85,86]. Trol/Perlecan is expressed by glial cells in close proximity to larval central brain neuroblasts, but not by the neuroblasts themselves [80]. Insulin-like peptides 2/6 (dILP2/6) production from these surface glial cells is sufficient to induce neuroblast reactivation through Akt signaling [80]. However, the authors note that the extent of reactivation by ectopic dILP2/6 is lower than endogenous levels of proliferation—they suggest that another nutrient-dependent mitogen is required for full reactivation [80]. Recently Kanai et al. (2018) demonstrated that expression of *Dally-like* (Dlp; a heparin sulphate proteoglycan) by perineural glia is required for continued neuroblast proliferation in the larval brain lobes by promoting *glass bottom boat* (Gbb; a BMP homologue) signaling within neuroblasts [87]. To test the role of glial-derived secreted factors in promoting larval neuroblast proliferation, the authors used a temperature sensitive allele of *shibire* (*shi-ts*). *shibire* is required for exocytosis and its loss of function at the restrictive temperature prevents the release of secreted factors by glia. Kanai et al. (2018) determined that inhibition of glial exocytosis with the *shi-ts* allele reduced neuroblast BrdU incorporation, confirming that glial-derived secreted factors promote neuroblast proliferation. In this study, the *shi-ts* allele was inactivated at the restrictive temperature prior to neuroblast reactivation (24 h after egg laying). Some component of the reduced neuroblast proliferation in these larvae may therefore be due to a lack of cell cycle re-entry of the post-embryonic neuroblasts, in addition to a loss of factors that promote continued proliferation following reactivation.

While these studies demonstrate the role of dILPs and other secreted factors in larval neuroblast proliferation, the mechanism through which the glycoprotein Ana functions is less clear. *ana* transcript was first described to be expressed specifically in glia [84], but a recent study detected *ana* transcription in neuronal populations marked by the *elav-GAL4* driver [88]. The authors also determined that the *ana* mRNA is a target of *miR-124*, introducing an additional layer to the regulation of neuroblast proliferation [88]. It remains unclear whether Ana functions purely non-autonomously on neuroblasts, and which population of cells is the relevant source of secreted Ana protein.

As described briefly above, the glia-neuroblast signaling axis in the larval CNS lies downstream of a yet-to-be-identified “fat body-derived mitogen”, which would connect incoming nutritional information from the digestive system of feeding larvae to dILP secretion by the insulin-producing cells within the central brain. Recent studies have identified fat body-secreted signals that induce insulin production, offering candidates for the fat body-derived mitogen (Figure 6C, reviewed in [89]). Delanoue et al. (2016) [90] determined that the secreted peptides SunA and SunB, gene products of *stunted*, are required for larval body growth and act through Methuselah, a GPCR found on insulin-secreting cells in the brain. Additionally, Koyama & Mirth (2016) [91] identified Growth Blocking Peptide (GBP) 1 and 2 as activators of insulin secretion. GBP1 was recently shown to physically interact with Methuselah-like receptor-10 (Mthl10; [92]), indicating a possible point of convergence of peptide signaling from the fat body to the Methuselah family of GPCRs in the brain. While these candidates are promising, their role in the reactivation of neuroblast proliferation has not yet been tested directly.

Neuroblast reactivation during larval development is ultimately dependent on the coupling of nutritional signals to the mitotic machinery to execute cell division. The recent observation that the majority of post-embryonic neuroblasts are arrested in G2 indicates that cell cycle re-entry of most larval neuroblasts occurs at the beginning of mitosis. Notably, *chromator* (Chro), a member of the spindle matrix complex, is essential for larval neuroblasts to respond to insulin/PI3K signaling in order to reactivate [93]. In addition to their role in the spindle matrix, both Chromator and its binding partner, East, associate with known transcriptional regulators and have been shown to modulate gene expression through their association with chromatin [94,95,96,97,98]. Notably, Chromator and East are required for expression of Grainyhead and repression of Prospero in larval central brain neuroblasts [93]. These observations could suggest a role for the spindle matrix complex to act as a signaling hub to integrate nutritional stimuli via insulin/PI3K and promote the execution of mitosis. Reactivation could potentially occur through direct interactions of the spindle matrix complex with other components of the kinetochore machinery, or through indirect transcriptional mechanisms. 

## 6. Control of Stem Cell Apoptosis

**Figure 7:**
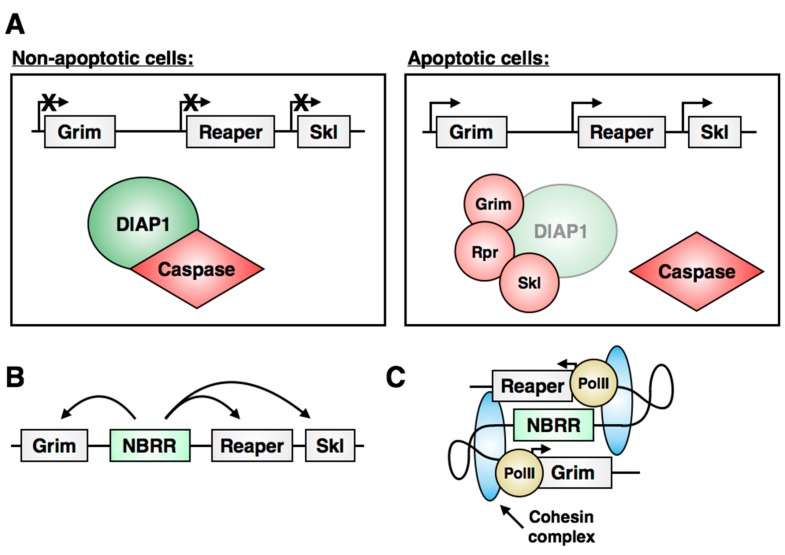
**Regulation of neuroblast apoptosis.** (**A**) The RHG genes *grim*, *reaper* and *sickle* are inactive in non-apoptotic cells, allowing DIAP1 to sequester and inhibit caspases. Apoptosis is induced following transcriptional activation of the RHG genes. RHG binding to DIAP1 promotes its turnover and releases caspases to allow their activation. (**B**) The intergenic enhancer NBRR is required for neuroblast apoptosis and for expression of the surrounding RHG genes. (**C**) A proposed model for regulation of RHG genes by long-range DNA interactions between the NBRR and RHG promoters mediated by the cohesin complex.

*Drosophila* has been a hugely informative model for the study of programmed cell death (PCD) for decades, since the genetic control of apoptosis was initially described in the early nineties [2,5,99,100]. Neuroblast apoptosis is induced by expression of the cell death genes *reaper (rpr)*, *grim* and *sickle (skl)*, members of the Reaper, Hid and Grim (RHG) gene family of apoptosis regulators [101,102]. These genes are clustered within the genome and are required in various combinations for virtually all embryonic cell death [2,101,102]. In non-apoptotic conditions, caspases are constitutively kept inactive by the *Drosophila Inhibitor of Apoptosis Protein* (DIAP1, Figure 7A left; [103,104,105]). RHG proteins bind to DIAP1 and competitively displace the bound caspases, leading to their cleavage and activation, while also promoting increased turnover of DIAP1 (Figure 7A right, [105,106,107,108,109,110,111]). A benefit of *Drosophila* as a model system for studying PCD is the stereotyped pattern of cell death during development, allowing investigations into the upstream regulatory pathways responsible for dictating when and where cell death is initiated *in vivo*. The combined mechanisms of spatial and temporal signals that regulate neuroblast apoptosis control cell fate with exquisite precision. The regulation and execution of apoptosis in *Drosophila* neuroblasts has been the subject of recent in-depth reviews [112,113].

Most developmental apoptosis of neuroblasts occurs in the abdominal segments of the embryonic nervous system: 27 of 30 abdominal neuroblasts within each hemisegment will die by apoptosis late in embryonic development. A small number of thoracic neuroblasts die at the same time, but little is known about the regulation of their death. A second wave of apoptosis occurs during the third instar larval stage and eliminates the remaining abdominal neuroblasts. Thus, abdominal neuroblasts have proven a useful model for the study of upstream factors that regulate the induction of apoptosis. Our lab has identified an intergenic enhancer within the RHG gene locus called the Neuroblast Regulatory Region (NBRR) that is required for embryonic and larval neuroblast apoptosis by regulating *grim*, *rpr* and *skl* through long-range interactions (Figure 7B; [102,114]). The NBRR is required for full expression of these genes, and an intergenic deletion that removes the NBRR (*Df(3L)MM3*) rescues many, but not all, neuroblasts from apoptosis [102]. As some death still occurs in these mutants, there are likely additional unidentified enhancer regions within the RHG locus that contribute to the execution of apoptosis. 

Perhaps unsurprisingly, the Hox gene *abdA*, specifically expressed in abdominal segments, has been shown to regulate neuroblast apoptosis at both embryonic and larval stages [3,20,114]. *abdA* is required for death of embryonic abdominal neuroblasts and is enough to induce apoptosis of thoracic neuroblasts. A reporter of NBRR activation is ectopically induced by *abdA-*misexpression, and this enhancer sequence was recently shown to be bound directly by AbdA in vitro [114,115]. Upstream of *abdA*, our lab showed that expression of the Notch ligand Delta by neural progeny is required for embryonic neuroblast death: progeny-induced Notch signaling within the neuroblast promotes AbdA expression and leads to activation of apoptosis through the NBRR [114]. The mechanism through which AbdA binding to the NBRR activates RHG gene expression remains unclear. We have recently shown that members of the cohesin complex are required for neuroblast death, consistent with a model of cohesin-mediated long-range interactions between the NBRR and the *rpr* and *grim* promoters (Figure 7C; [116]). In addition, we determined that the DNA-binding protein Cut is required for apoptosis, and loss of Cut protein is associated with an increase in the repressive heterochromatin mark H3K27me3 within the RHG locus, and globally in neuroblasts, during embryogenesis [116]. These findings support a role for chromatin architecture and long-range DNA interactions in the transcriptional regulation of the RHG genes. 

While Hox genes determine the spatial dimension of apoptosis induction, the timing of embryonic abdominal neuroblast apoptosis is controlled by the temporal series. There is a fundamental link between the molecular clock of the temporal series and neuroblast fate: the transcription factors *Dichaete* (D) and *grainyhead* (Grh) both regulate apoptosis and are targets of the late embryonic temporal series factor Castor (see Figure 4A; [4,117,118]). Specifically, Grh is required for apoptosis and has recently been shown to bind to the NBRR [115], while Dichaete misexpression leads to ectopic neuroblast survival [4]. These results suggest that upon completion of the embryonic temporal series and expression of Cas, loss of D expression and gain of Grh expression would activate the NBRR and lead to RHG gene expression. However, Grh expression is constitutive in many embryonic neuroblasts that do not undergo apoptosis [118]. In addition, as the temporal series is shared across the thorax and abdomen, it is unclear how Grh expression leads to abdomen-specific induction of apoptosis. The most obvious explanation would involve combinatorial activity of Grh with Hox genes, leading to terminal division in the thorax and apoptosis in the abdomen. However, this model has not yet been directly tested, and the differences in Grh/Hox gene downstream targets across different regions of the VNC are unknown. In addition, the apoptosis of a small number of thoracic neuroblasts indicates additional levels of neuroblast-specific regulation of cell death, even within a given anatomical region.

## 7. Regulation of Terminal Division

While both embryonic and post-embryonic abdominal neuroblasts are eliminated by apoptosis, the post-embryonic thoracic and central brain neuroblasts undergo a terminal division in the larval CNS (see Figure 3A). The final symmetrical division of these neuroblasts is induced by nuclear accumulation of Prospero, which leads to down-regulation of self-renewal genes and promotes differentiation [4,77]. Similar to the apoptotic fate decision in the abdominal post-embryonic neuroblasts, terminal division of post-embryonic thoracic neuroblasts lies downstream of the temporal series. In *castor* and *seven-up* mutants, which do not complete the temporal series, larval neuroblasts in the central brain and thorax continue to proliferate during adulthood [4]. However, it was recently reported that loss of Castor within the NB5-6 lineage did not result in changes in Prospero expression or localization during mid-embryogenesis [119]. It remains to be tested whether this reflects lineage-specific responses to changes in the temporal series, or whether embryonic and post-embryonic neuroblasts differ in their sensitivity to the induction of nuclear Prospero. 

**Figure 8:**
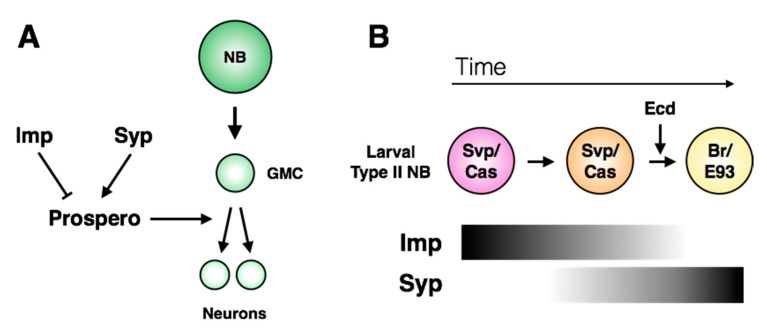
**Regulation of neuroblast terminal division.** (**A**) The RNA-binding proteins Imp and Syp have opposing effects on Prospero-mediated terminal division. Imp is sufficient to prevent cell cycle exit, but Syp may play an indirect permissive role for Prospero function. (**B**) Imp and Syp expression correlates to progression of the larval temporal series. Imp expression is down-regulated following the pulse of ecdysone, but Syp expression begins prior to this pulse.

Downstream of the temporal series, *hedgehog* (Hh) signaling is necessary and sufficient for cell cycle exit of larval central brain neuroblasts. Hh expression is promoted by Cas, and leads to nuclear Prospero accumulation and mitotic exit [120]. A more recent study implicated RNA-binding proteins *IGF-II mRNA-binding protein* (Imp) and *Syncrip* (Syp) in the regulation of Prospero localization in central brain neuroblasts (Figure 8A; [121]). The expression patterns of Imp and Syp vary through development: Imp is co-expressed with the early larval factors Castor, Seven-Up and Chinmo, while Syp is expressed with the late factors Broad and E93 (Figure 8B; [26,27,122]). *pros* mRNA was identified as a target of Syp by RNA-immunoprecipitation, suggesting a direct role in regulating Prospero protein levels [123]. While Imp is sufficient to inhibit terminal division in the larval CNS, Syp misexpression does not induce Prospero protein accumulation or early terminal division, indicating that it may act permissively rather than directly [121]. The molecular details that connect Imp/Syp and temporal signaling to the Hedgehog pathway remain unknown. 

Much focus has been placed on the transcription factor networks that regulate stem cell decisions, but, as seen with neuroblast reactivation, metabolic needs also influence proliferation of stem cells. The mushroom body neuroblasts, which continue to proliferate for 96 h in the pupal brain, are finally eliminated by apoptosis induced by insulin/PI3K signaling [124]. Similarly, the terminal division of larval central brain neuroblasts is driven by metabolic changes within the stem cell: release of the steroid hormone ecdysone promotes metamorphosis and pupariation, while inducing a switch in neuroblast growth mode [32]. Ecdysone signaling causes larval central brain neuroblasts to undergo “shrinkage”, where each asymmetric division is accompanied by a reduction in neuroblast cell volume. In an unbiased screen, the authors identified the Mediator complex and oxidative phosphorylation (OxPhos) machinery as drivers of reductive neuroblast divisions. Loss of Mediator complex members, ecdysone signaling or OxPhos components all prevented shrinkage and increased neuroblast proliferation [32]. Similar to the central brain neuroblast shrinkage, reductive divisions are observed in embryonic neuroblasts [125]. However, these do not result in terminal division in the embryo, and it is not known whether embryonic shrinkage is ecdysone-dependent. In addition, it remains to be determined how the OxPhos switch is linked to Prospero localization. 

Interestingly, terminal division and apoptosis seem to be mutually exclusive fates: apoptosis-deficient larval neuroblasts (homozygous for deletion of the RHG genes) display continuously proliferating abdominal neuroblasts [3]. This finding suggests that larval abdominal neuroblasts do not accumulate nuclear Prospero prior to their death. However, it remains unclear whether these ectopic abdominal neuroblasts continue to proliferate indefinitely, as terminal division of thoracic and central brain neuroblasts occurs more than 24 h later than larval abdominal neuroblast apoptosis, and this later time period was not examined in the study by Bello et al (2003) [3]. It is, therefore, unclear whether cell death-defective abdominal neuroblasts are competent to respond to systemic ecdysone signaling by undergoing terminal division, or whether this fate decision is truly restricted to larval neuroblasts of the thorax and brain. 

## 8. Misregulation of Stem Cell and Progeny Identity 

The previous sections have discussed endogenous differences in stem cell proliferation patterns across the *Drosophila* CNS, as well as the regulatory inputs that control quiescence and cell cycle re-entry. Throughout these dynamic changes, stem cell identity must be tightly regulated. Loss of stemness would inhibit tissue growth and could lead to hypoplasia, while gain of stemness in progeny cells could lead to uncontrolled proliferation and a tumor-like state. A potent inducer of the neuroblast identity is Notch: overexpression of the active Notch intracellular domain (N^icd^) leads to massive tissue overgrowth and an increase in the number of neuroblasts at the expense of differentiated progeny [114,126,127,128]. The *E(spl)/HES* genes, downstream targets of Notch, are both necessary and sufficient for larval neuroblast over-proliferation [128,129]. While this effect of Notch gain-of-function has been well characterized in larval neuroblasts, the output of the Notch signaling pathway is context-dependent. As discussed previously, Notch signaling in the embryonic neuroectoderm inhibits neuroblast formation [6], and Notch promotes the Type I > 0 switch at the end of embryogenesis [119,130]. These differential effects of Notch in neuroblasts highlight the extent to which this signaling pathway is integrated with spatial and temporal inputs throughout development.

**Figure 9:**
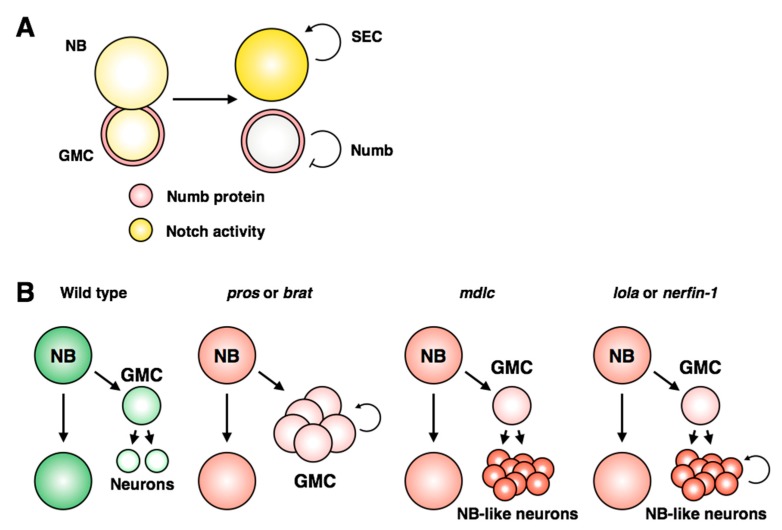
**Misregulation of stem cell and differentiated cell identity.** (**A**) Notch activity is detected equally in the stem cell and GMC following neuroblast division, but quickly becomes restricted to the neuroblast [128]. The super elongation complex (SEC) promotes a positive feedback loop with Notch signaling in the neuroblast, while Numb inhibits Notch specifically in the GMC. (**B**) Loss of differentiated cell identity can lead to unchecked proliferation in the nervous system. Two classes of dedifferentiation mutants have been identified: *pros* and *brat* mutants fail to restrict the GMC to a single terminal division, while *mdlc*, *lola* or *nerfin-1* mutants display ectopic expression of neuroblast markers in neurons. *lola* and *nerfin-1* mutants also result in ectopic proliferation of neuronal cells.

In larval neuroblasts, the Notch inhibitor Numb is asymmetrically transmitted to the GMC during mitosis to prevent activation of Notch targets in progeny cells (Figure 5A; [131,132,133]). However, a recent examination of live larval neuroblast cell divisions showed that Notch activity is detected equally in the neuroblast and daughter GMC for up to 40 minutes after division [128]. The authors determined that the super elongation complex (SEC) acts as a signal amplifier to strengthen Notch activity within the neuroblast: the SEC promotes expression of *E(spl)/HES* genes, which in turn promote expression of SEC members (Figure 9A). This model provides direct mechanistic evidence for how Notch activity is restricted to, and amplified within, neuroblasts. 

Interestingly, the Notch targets *E(spl)* and *dpn* are required to maintain neuroblast identity through the post-embryonic quiescence period [129], but the molecular details are not clear. It was very recently reported that the v-ATPase complex, an intracellular transmembrane proton pump, and a v-ATPase-interacting protein Vap33, are required for Notch signaling and neuroblast regrowth following quiescence [134,135]. v-ATPase and Notch form a feedback regulatory loop [134], reminiscent of the SEC-Notch signaling connection. These examples highlight the central role of Notch signaling in neuroblast identity and offer a common model of regulatory positive feedback through which this identity can be maintained. 

Loss of cell identity in differentiated cells can lead to a gain of stem-like behavior, associated with ectopic proliferation and loss of differentiation markers. A prominent example is the phenotype resulting from *prospero* loss of function, which leads to massive overgrowth of the nervous system due to lack of repression of cell cycle genes in GMCs and neurons [77]. This phenotype is consistent with an absence of differentiated cell identity, indicating that transmission of Prospero to daughter cells is necessary for the neuronal and/or glial cell identity program. In Type II neuroblast lineages, the INP identity must also be restricted with respect to the parental neuroblast, to prevent dedifferentiation back into a stem cell. The phenotype of *brat* mutants, which show unrestrained proliferation of daughter cells, is consistent with loss of INP identity and gain of stemness [136,137]. Brat promotes INP identity by antagonizing the transcription factor Klumpfuss, which is sufficient to convert INPs into neuroblasts [138]. Multiple additional regulators have been identified that are required for the restriction of INP identity: the transcriptional repressor Earmuff, the EGF signaling component PntP1, the transcription factor Buttonhead and multiple members of the SWI/SNF complex have all been implicated in preventing loss of the INP identity [59,139,140]. 

In contrast to these examples, another class of mutants has been identified in which *bona fide* dedifferentiation of neurons occurs (Figure 9B; reviewed in [141]). Neuronal dedifferentiation has been described following loss of function of each of the zinc finger-containing proteins *midlife crisis* (Mdlc), *longitudinals lacking* (Lola) and *nervous fingers 1* (Nerfin-1) [142,143,144]. While Mdlc was found to regulate Prospero expression through splicing [142], Nerfin-1 promotes neuronal differentiation in a Prospero-independent manner [144]. Lola shares many target genes with Prospero [143], suggesting that these genes may act redundantly. However, GMCs are able to differentiate properly in *lola* mutants, but not *pros* mutants, indicating that they are functionally distinct in the context of differentiation (Figure 8B; [143]). It remains unclear how these three examples cooperate to retain the differentiated state, or if there is cross-regulation between the pathways. Lola and Nerfin-1 proteins are excluded from neuroblasts, but Midlife Crisis is expressed broadly throughout the nervous system, indicating that neural stem cells use distinct mechanisms to repress the activity of these regulators of neuronal identity to prevent premature differentiation of the progenitor pool.

## 9. Concluding Thoughts

The concept of stem cell identity has in recent years shown increasing granularity: the advent of single-cell transcriptomics has unveiled the vast amount of heterogeneity within populations of cells previously thought to be homogenous. The *Drosophila* CNS offers a complex but experimentally amenable model for investigating the regulation of stem cell behavior. Populations of neural stem cells throughout the CNS have in common the ability to generate neural progeny, and they express many of the same molecular markers. Despite these shared features, their identities and cell-fate decisions are fundamentally distinct. The pathways and networks shared by *Drosophila* neural stem cells have been well characterized, providing a stepping-stone to begin to tease out the differences inherent to these populations. The genetic tools made available within the fly community, notably from the Janelia FlyLight Project [145,146,147,148], now allow identification and manipulation of specific neuroblasts *in vivo*. These tools offer an unprecedented opportunity for investigations into the endogenous heterogeneity of stem cell behavior and highlight the power of *Drosophila* as a model system.
